# Proteomic Analysis of Embryo Isolated From Mature *Jatropha curcas* L. Seeds

**DOI:** 10.3389/fpls.2022.843764

**Published:** 2022-03-18

**Authors:** Ayesha Ramzan, Mohibullah Shah, Najeeb Ullah, Sheheryar, José R. S. Nascimento, Francisco A. P. Campos, Gilberto B. Domont, Fábio C. S. Nogueira, Magda H. Abdellattif

**Affiliations:** ^1^Department of Biochemistry, Bahauddin Zakariya University, Multan, Pakistan; ^2^Department of Biochemistry and Molecular Biology, Federal University of Ceara, Fortaleza, Brazil; ^3^Department of Biochemistry, Institute of Chemistry, Federal University of Rio de Janeiro, Rio de Janeiro, Brazil; ^4^Laboratory of Proteomics/LADETEC, Federal University of Rio de Janeiro, Rio de Janeiro, Brazil; ^5^Department of Chemistry, College of Science, Taif University, Taif, Saudi Arabia

**Keywords:** *Jatropha curcas*, subproteome, embryo, biodiesel, oilseed

## Abstract

*Jatropha curcas* L. is a non-edible oilseed containing almost 40% of seed oil and is famous as the best source of raw material for biofuel production. *J. curcas* seeds contain three main tissues, such as inner integument, endosperm, and embryo. To best understand the physiological events related to specific tissues, it is important to perform the proteome analysis of these tissues. Previously we have explored the pattern of reserves deposition and tissue-specific biological pathways by analyzing the proteome of the inner integument and endosperm and organelles, such as plastids and gerontoplasts isolated from these tissues. The focus of the present study was to perform the proteomic analysis of embryo isolated from the mature seeds of *J. curcas.* This analysis resulted in the identification of 564 proteins of which 206 are not identified previously from any other tissue of this plant. The identified proteins were functionally classified using the MapMan classification system revealing various proteins involved in different functionalities. The proteins involved in transport functions and those with proteolytic activity were determined through the Transporter Classification Database (TCDB) and MEROPS database, respectively. In addition to identify a large number of proteins participating in various metabolic processes, we found several proteins involved in defense functions, such as the members of chaperones and the ubiquitin-proteasome system. Similarly, members of the legumin and vicilin family of seed storage proteins (SSPs) were identified which in addition to their storage function, are involved in defense. In addition, we have reported that proteases belonging to different mechanistic classes and are involved in diverse physiological functions. Last but not the least, several classes of transport-related proteins were identified that are discussed concerning their function in the transportation of different nutrients across the embryo. To the best of our knowledge, this study reported the highest number of proteins identified from the embryo of mature *J. curcas* seeds, most of which are essential for seed germination, reflecting the fact that many proteins required for germination are already present in the mature embryo.

## Introduction

The seeds of embryonic plants are comprised of three basic components, an embryo, embryo proper tissue, and a seed coat to insulate seed from environmental stress (De Smet et al., 2010). Nutrients can be stored in various tissues, such as cotyledons, endosperm, or megagametophyte. In a few seeds, an embryo is trapped in the endosperm, and in other cases, the endosperm is absorbed by the embryo. In the latter case, the embryo develops inside the growing seeds, and cotyledons become enriched with the storage products (Miernyk et al., 2011). *Jatropha curcas* L. is a non-edible oil rich shrub and is best known for its potential to be used as a feedstock for biodiesel production. The key reasons are its oil richness, rapid growth, drought resistance ability, and necessary adaptation to widespread environmental conditions. Mature *J. curcas* seeds contain a tiny embryo enclosed in a dense endosperm (Liu et al., 2009). Both embryo and endosperm have distinct ploidies and interact in a coordinated manner to control the seed growth.

The availability of the draft genome sequence of *J. curcas* enabled the scientific community to perform sub-proteome analysis associated with the seed development. In this aspect, our first study was focused on plastids (Pinheiro et al., 2013) isolated from the endosperm of mature seeds resulting in the identification of 923 unique proteins associated with the biosynthesis of amino acids and fatty acids. This study was followed by the comparative proteome analysis of the two distinct regions of the inner integument in which a total of 1,770 proteins were identified (Soares et al., 2014). This study highlighted the role of different enzymes, i.e., proteases, nucleases, lipases, and carbohydrate-acting enzymes in programmed cell death (PCD), which are associated with the provision of nutrients for growing embryo and endosperm. Though these two studies provided insight into the diverse biological aspects of the seeds, the changes associated with the proteome during seeds development remained unanswered. To fill this gap, a comprehensive proteomic analysis of the endosperm at five different developmental stages was performed (Shah et al., 2015). The major classes of 1,760 identified proteins were those, related to seed storage, lipids and carbohydrates metabolism, xenobiotics metabolism, and proteolysis. Moreover, this study revealed the deposition of various isoforms of these proteins during different developmental stages of the seed. To determine the biological aspects of the plastids transition to gerontoplast, we had performed their histological and proteomic investigations. For this purpose, the plastids were isolated from the inner integument of developing *J. curcas* seeds and identified 1,923 proteins after proteomic analysis (Shah et al., 2016). Here, we reported and discussed proteins involved in a myriad of functions related to the dismantling of this tissue, providing nutrients to other tissues under development. Despite the importance of the embryo in the seeds, its proteome is yet unanalyzed to reveal the biological pathways associated with this tissue in *J. curcas* seeds. Previously two different studies utilized the two-dimensional gel electrophoresis (2DE) based approach for analyzing the embryo proteome of *J. curcas* seeds (Liu et al., 2009, 2011). However, these studies collectively identified < 30 proteins, most of which were related to reserve mobilization during germination.

In hitherto studies, we have targeted various tissues and organelles of *J. curcas* which collectively resulted in the identification of 6,188 proteins of unique physiological characteristics. In this regard, the current study is aimed to analyze the proteome of an embryo isolated from mature *J. curcas* seeds to disseminate biological pathways related to this important tissue. Here, we obtained 564 proteins with 206 newly identified and were never reported in our previous results from other tissues. The identified proteins were discussed with emphasis on their involvement in seed development and reserves deposition and mobilization during germination.

## Materials and Methods

### Embryo Isolation and Protein Extraction

**FIGURE 1:**
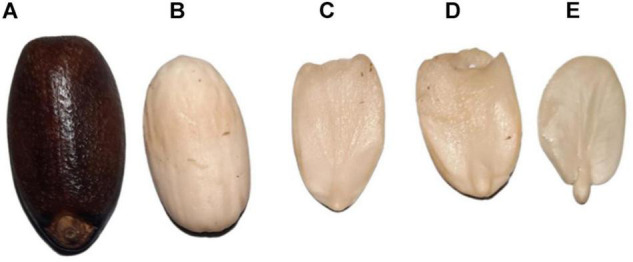
The mature seed of *Jatropha curcas.* **(A)** Mature seed with seed coat. **(B)** Mature seed without a seed coat. **(C,D)** Endosperm. **(E)** Embryo.

Mature *J. curcas* seeds were collected from the Punjab Province of Pakistan. Seeds were manually dehulled and the embryos were separated from seeds (Figure 1) with a scalpel to prevent its contact with endosperm and inner integument. To remove the lipids, the embryos were cut into small fragments and placed under gentle stirring in acetone for almost 30 h. The acetone was changed every 5 h. This material was then dried at room temperature and ground into powder with mortar and pestle by using liquid nitrogen. The powdered material was then stored at −80°C until further use. Powdered embryos were subjected to protein extraction following a previously established method (Vasconcelos et al., 2005). For the extraction of soluble proteins, 3 replicates of 0.1 g powdered embryo were weighed and homogenized in 5 ml of pyridine buffer (50 mM pyridine, 10 mM thiourea, and 1% SDS, pH 5.0) with polyvinyl-polypyrrolidone in a ratio of 1:40:2 (w/v/w). The mixture was kept at stirring for 3 h at 4°C. The centrifugation was performed for 30 min at 10,000 rpm. Proteins were precipitated from the supernatant using trichloroacetic acid (10%) in acetone. Cold acetone was used to wash the pellets three times, centrifuged, and dried at room temperature. The dried pellets were solubilized in sample buffer containing 7 M urea/2 M thiourea and 100 mM triethylammonium bicarbonate (TEAB) buffer. The same process was repeated for all three biological replicates. Bradford assay (Bradford, 1976) was used to measure the concentration of proteins using bovine serum albumin (BSA) as standard.

### Samples Preparation for Liquid Chromatography With Tandem Mass Spectrometry (LC-MS/MS)

In-solution trypsin digestion was carried out with 50 μg of proteins following our previously used protocol (Pinheiro et al., 2013). Before trypsinization, the proteins were reduced using 10 mM tris (2-carboxyethyl) phosphine (TCEP) for 1 h at 30°C and alkylated with 40 mM iodoacetamide at room temperature in the dark for 30 min. To reduce the urea concentration to less than 1 M, the samples were diluted with 100 mM TEAB (1:9 v/v). Finally, samples were subjected to trypsin digestion for 18 h at 37°C. Proteins digestion was quenched with TFA. After digestion, the resulting peptides were cleaned through a spin column, dried in speed-vacuum (SpeedVac), and stored at −80°C for further use.

### Nanoscale Liquid Chromatography Coupled to Tandem Mass Spectrometry (NanoLC-MS/MS) and Data Analysis

Before the introduction to nanoscale liquid chromatography coupled to tandem mass spectrometry (NanoLC-MS/MS), peptides were solubilized in 20 μl of 0.1% formic acid and diluted to 5x. These peptides were quantified with a Qubit protein assay kit. Diluted samples (4 μl) were subjected to NanoLC-MS/MS system interfaced online to ESI-LTQ Orbitrap Velos MS. Peptides were loaded onto a 150 μm × 2 cm trap column packed with C-18 ReproSil 3 μm resin and then eluted onto an analytical column of 100 μm × 15 cm packed with the similar resin. The separated peptides were collected through a gradient from 100% of A (0.1% formic acid) to 35% of B (0.1% formic acid and 95% acetonitrile) for 150 min, followed by 35–90% of solution B for 15 min and 90% for 5 min. For MS1 spectra, each data-dependent acquisition mode comprised of a survey scan covering a range of m/z 300–2,000 and 60,000 resolution with a targeted value of 1 × 10^–6^ ions. Tandem mass spectrometry (MS/MS) fragmentation of the ten major intense ions was acquired using a normalized collision-induced dissociation of formerly fragmented ions. The m/z of fragmented precursor ions were excluded for 60 s. Each biological replicate was injected three times which resulted in nine technical replicates for three biological replicates of the sample.

An Xcalibur v.2.1 (Thermo Fisher Scientific) was used to view the raw files while database search was performed using the Sequest™ algorithm embedded in Proteome Discoverer 2.1 (Thermo Fisher Scientific) against the combined database of *J. curcas* nuclear (Chan et al., 2010) and plastid (Asif et al., 2010) genomes. The search parameters were: MS accuracy 10 ppm, MS/MS accuracy 0.1 Da, trypsin digestion with two missed cleavages, carbamidomethylation of cysteine as fixed oxidized methionine as variable modification. A false discovery rate of 1% was used at the protein and peptide level. The identified proteins of *J. curcas* embryo that appeared in almost 2 biological replicates were used for the downstream analysis. For annotation of the identified proteins, we performed a local BLAST of the identified proteins against *Arabidopsis thaliana* and *Ricinus communis* protein databases that were downloaded from TAIR^1^ and UniProt,^2^ with *e*-value of 1 × 10^–5^ for determining the respective orthologous proteins. Protein functional classification was performed according to the bincodes of MapMan.^3^ For the classification of proteases, identified proteins were scanned against MEROPS database (Rawlings et al., 2018)^4^ using blastp with an *e*-value of 1 × 10^–15^. For the classification of transporters, TCDB (Saier et al., 2016)^5^ was used with an *e*-value of 1 × 10^–15^.

## Results and Discussion

### Proteins Identification and Functional Classification

**FIGURE 2:**
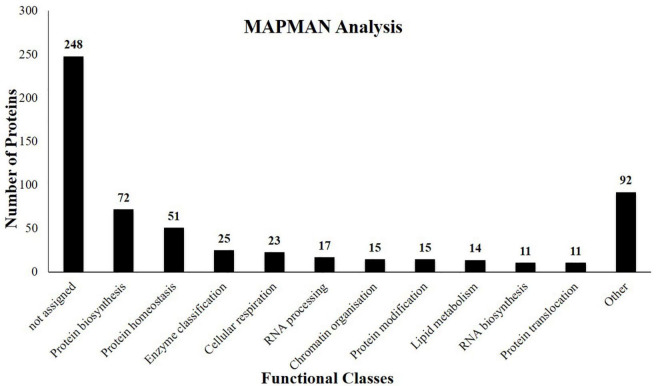
Functional classes of the proteins identified from the embryo of *J. curcas* seeds based on the bincodes of MapMan.

Proteomic studies on *J. curcas* resulted in a wealth of information regarding biological events associated with various tissues of the seeds. However, limited attention was given to the embryo. In this study, we have identified 564 proteins (Supplementary Table 1) from the *J. curcas* embryo of which 208 were previously not identified from other tissues of this plant. The identified proteins were functionally classified using a MapMan classification system (Schwacke et al., 2019). The major functional classes include protein metabolism, lipid metabolism, carbohydrate metabolism, defense-related proteins, and proteases among the others (Figure 2).

### Lipid Metabolism Related Proteins

*Jatropha curcas* is an industrially significant oilseed crop mainly due to the high quantity of oil in their seeds. Although in *J. curcas* seeds, endosperm is the main site for the oil accumulation, however, lipids are also present in the embryo (Chen et al., 2011; de Lopes et al., 2013). In the present study, 27 lipid metabolism-related proteins were identified (Table 1) corresponding to almost 5% of the total identified proteins and are involved in the numerous metabolic pathways of lipids. The initial phase of this pathway relies on acetyl-CoA for the biosynthesis of FA in plastids. Acetyl-CoA is provided by the plastidial pyruvate dehydrogenase complex, of which several subunits are identified here. Similarly, acetyl-CoA carboxylase, a biotin-bound one carbon carrier protein (Jcr4S01232.50) catalyzes the abridgment of acetyl-CoA to malonyl-CoA, is also identified. In addition, we identified ketoacyl-ACP synthase I (Jcr4s02541.50) catalyzing the synthesis of 18 carbon FA *via* condensation reactions. A series of desaturation reactions introduce desaturation and play a significant role in lipids quality (Gu et al., 2012). Previously, transcripts for multiple desaturase proteins, e.g., stearate, oleate, and linoleate desaturase were identified from *J. curcas* seeds (Costa et al., 2010). Significantly, four isoforms of stearate desaturase (Arabidopsis homologous) were identified from the endosperm proteome of *J. curcas* seeds (Shah et al., 2015). However, in the present study, we did not identify any desaturase from the embryo of *J. curcas* seeds. The possible reason might be due to lower identifications as compared with our previous studies.

**TABLE 1:** Proteins related to lipid metabolism identified from the embryo of mature *Jatropha curcas* seeds.

Protein accession	Description	Best hit *Ricinus*	Best hit *Arabidopsis*
Jcr4S01232.50	Acetyl-CoA carboxylase biotin carboxyl carrier protein	B9RM56	AT5G16390.1
Jcr4S02541.50	Ketoacyl-ACP synthase I	Q41135	AT5G46290.1
Jcr4S00031.240	3-Oxoacyl-acyl-carrier protein reductase	B9SHH2	AT4G13180.1
Jcr4S00575.60	Pyruvate dehydrogenase E1 component alpha subunit	B9S2H9	AT1G59900.1
Jcr4S01924.60	Dihydrolipoamide acetyltransferase component E2	B9SL87	AT3G52200.1
Jcr4S00306.100	Dihydrolipoamide acetyltransferase component E2	B9S5V2	AT1G54220.2
Jcr4S04485.30	Dihydrolipoamide succinyltransferase component E2	B9SVA1	AT4G26910.1
Jcr4s00112.160	Dihydrolipoamide dehydrogenase component E3	B9RZN2	AT3G16950.1
Jcr4S00609.30	Alcohol dehydrogenase	B9R8J1	AT1G22440.1
Jcr4U29909.10	Alcohol dehydrogenase	B9R8J1	AT1G22440.1
Jcr4S02965.20	Alcohol dehydrogenase	B9SJJ8	AT1G77120.1
Jcr4S00529.80	Alcohol dehydrogenase	B9SJJ8	AT1G77120.1
Jcr4S03546.10	Aldehyde dehydrogenase	B9RB49	AT3G48000.1
Jcr4s05992.20	Oleosin	Q5VKJ9	AT3G01570.1
Jcr4s01276.90	Oleosin	Q5VKJ8	AT4G25140.1
Jcr4s01398.30	Caleosin (Calcium ion binding protein)	B9SQU8	AT4G26740.1
Jcr4s01018.60	3-Ketoacyl-CoA thiolase (KAT)	B9RWL7	AT2G33150.1
Jcr4s09697.10	Aconitase	B9SXB6	AT4G35830.1
Jcr4s00736.30	Aconitase	B9T2U5	AT2G05710.1
Jcr4s00100.200	Malate synthase	B9RAK0	AT5G03860.2
Jcr4S00086.110	Phospholipase D1/2	B9RV56	AT1G52570.1
Jcr4S01462.20	Chloroplastic oxoene reductase	B9TA76	AT4G13010.1
Jcr4s00279.20	NAD-dependent malate dehydrogenase	B9T5E4	AT5G43330.1
Jcr4s03576.70	NADP-dependent malic enzyme	B9RS07	AT5G25880.1
Jcr4s03356.50	NADP-dependent malic enzyme	B9RKI6	AT5G25880.1
Jcr4s03281.10	NADP-dependent malic enzyme	B9RQE8	AT1G79750.1

Fatty acids are condensed with glycerol skeleton to form triacylglycerols (TAGs) and finally aggregate in the form of oil bodies (OBs) inside the endoplasmic reticulum (ER). OBs consist of a core of TAGs accompanied by various proteins, such as oleosins and caleosins (calcium-binding peroxygenases) incorporated in a phospholipid monolayer. In this study, we have identified two isoforms of oleosins (Jcr4s05992.20 and Jcr4s01276.90) and one caleosin (Jcr4s01398.30). In addition to these two oleosins, three other isoforms of oleosins were reported from our previous study on *J. curcas* endosperm (Shah et al., 2015). This may indicate the fact that different isoforms of the same proteins are active in different tissues. Oleosins are responsible for altering the OBs size and lipid accumulation in different seed tissues of *Arabidopsis*. Such storage deposits are typically large insoluble compounds that can remain stable for long spells in desiccated seeds (Siloto et al., 2006). Our results suggest that in *J. curcas* seeds embryo, oleosins might be involved in stabilizing OBs during the seed desiccation phase. This might prevent their degradation until they are needed for germination.

We have identified phospholipase D1 (Jcr4S00086.110) responsible for the large-scale breakdown of lipids related to cell death (Shah et al., 2015) and most often contributes to caspase dependent cellular death signaling mechanism. Previously we found them in the inner integument (Soares et al., 2014) and endosperm (Shah et al., 2015) of *J. curcas* seeds along with other hydrolases. They were believed to be involved in PCD in these different tissues. A previous study showed that the enhanced activity of proteins involved in β-oxidation and the glyoxylate pathway resulted in the deposition of particular fatty acids in growing rapeseeds (Chia et al., 2005). Here, we identified two glyoxylate cycle enzymes including two isoforms of aconitase (Jcr4s09697.10 Jcr4s00736.30) and one of malate synthase (Jcr4s00100.200). Acetyl CoA (end product of β-oxidation) serves as a substrate for the glyoxylate cycle (a modified form of TCA cycle) that skips the decarboxylation phase and permits the net production of carbon skeletons (succinate) without carbon loss as CO_2_ (Cornah and Smith, 2002). These findings suggest that during seed germination, the stored lipids will be degraded through the successive operation of β-oxidation, lipoxygenase, and glyoxylate cycle to provide carbon and energy for the seedling growth.

### Carbohydrate Metabolism Related Proteins

We identified 51 carbohydrate metabolism-related proteins (Table 2) correspond to 9% of the total identifications. In oilseeds, reserves are primarily synthesized during the seed-filling phase characterized by alterations in the morphological, cellular, and metabolic processes of embryo and endosperm (Hill et al., 2003). *De novo* synthesis of lipids requires carbon, energy, and reducing equivalents and is provided directly or indirectly *via* glycolysis in the form of acetyl-CoA, ATP, and nicotinamide adenine dinucleotide phosphate (NADPH) (Plaxton and Podestá, 2006). In the present study, several cytosolic and plastidial isoforms of glycolytic proteins were identified (Table 2). We identified fructose-1,6-bisphosphate aldolase (FBA) (Jcr4S14120.10) that catalyzes the aldol cleavage of Fru-1,6-bisP to glyceraldehydes-3-P (GAP) and dihydroxyacetone phosphate (DHAP). A previous research indicated that in heterotrophic embryos of sunflower, triose-phosphates were the primary source of carbon for the biosynthesis of fatty acids, but did not find the source of triose-phosphate production (Alonso et al., 2007).

**TABLE 2:** Proteins related to carbohydrate metabolism identified from the embryo of mature *J. curcas* seeds.

Protein accession	Description	Best hit *Ricinus*	Best hit *Arabidopsis*
Jcr4S00433.130	Phosphofructokinase	B9RXE7	AT1G12000.1
Jcr4S14120.10	Fructose-bisphosphate aldolase	B9SRH4	AT2G36460.1
Jcr4S02610.20	Fructose-bisphosphate aldolase	B9T5T6	AT2G36460.1
Jcr4S01786.40	Fructose-bisphosphate aldolase	B9SJY9	AT2G01140.1
Jcr4S00484.40	Fructose-bisphosphate aldolase	B9S0W4	AT4G26530.2
Jcr4S07385.10	Glyceraldehyde-3-phosphate dehydrogenase	B9RNW8	AT2G24270.3
Jcr4S00953.40	Glyceraldehyde-3-phosphate dehydrogenase	B9RNW8	AT2G24270.3
Jcr4S00273.150	Glyceraldehyde 3-phosphate dehydrogenase	B9RAL0	AT3G04120.1
Jcr4S00205.140	Glyceraldehyde 3-phosphate dehydrogenase	B9RAL0	AT1G13440.1
Jcr4S00043.160	Phosphoglycerate kinase	B9RHY3	AT1G79550.2
Jcr4s28329.10	Phosphoglycerate mutase	B9RF09	AT3G05170.1
Jcr4S00171.20	Phosphopyruvate hydratase (enolase)	B9R9N6	AT2G36530.1
Jcr4S00445.90	Phosphoglucomutase	B9SP64	AT1G70730.3
Jcr4S00575.60	Pyruvate dehydrogenase E1 component alpha subunit	B9S2H9	AT1G59900.1
Jcr4S01924.60	Dihydrolipoamide acetyltransferase	B9SL87	AT3G52200.1
Jcr4S00306.100	Dihydrolipoamide acetyltransferase	B9S5V2	AT1G54220.2
Jcr4S04485.30	Dihydrolipoyllysine-residue succinyltransferase	B9SVA1	AT4G26910.1
Jcr4S00112.10	Dihydrolipoamide dehydrogenase	B9RZN2	AT3G16950.1
Jcr4S08285.10	Phosphoenolpyruvate carboxykinase	B9R6Q4	AT4G37870.1
Jcr4S16847.20	6-Phosphogluconolactonase	B9RWU5	AT5G24400.1
Jcr4S00057.90	Transketolase	B9RDA1	AT2G45290.1
Jcr4S00380.50	Aldose 1-epimerase	B9SYV8	AT3G17940.1
Jcr4S00100.200	Malate synthase	B9RAK0	AT5G03860.2
Jcr4S03576.70	Malic enzyme	B9RS07	AT5G25880.1
Jcr4S03356.50	Malic enzyme	B9RKI6	AT5G25880.1
Jcr4S03281.10	Malic enzyme	B9RQE8	AT1G79750.1
Jcr4U29909.10	Alcohol dehydrogenase	B9R8J1	AT1G22440.1
Jcr4S00609.30	Alcohol dehydrogenase	B9R8J1	AT1G22440.1
Jcr4S00529.80	Alcohol dehydrogenase	B9SJJ8	AT1G77120.1
Jcr4S02965.20	Alcohol dehydrogenase	B9SJJ8	AT1G77120.1
Jcr4S03546.10	Aldehyde dehydrogenase (NAD +)	B9RB49	AT3G48000.1
Jcr4S03703.10	Aminobutyrate aminotransferase, putative	B9S4Y5	AT3G22200.1
Jcr4S02325.10	(1– > 3)-beta-glucan endohydrolase	B9SP53	AT2G01630.1
Jcr4S02004.20	Phosphoprotein phosphatase, putative	B9RYC0	AT3G19420.1
Jcr4S01159.50	Catalase	B9R8N4	AT4G35090.1
Jcr4S00684.10	UTP–glucose-1-phosphate uridylyltransferase	B9SKS5	AT5G17310.2
Jcr4S00313.50	Glucan endo-1,3-beta-glucosidase	B9T103	AT1G66250.1
Jcr4S09697.10	Aconitate hydratase (Aconitase)	B9SXB6	AT4G35830.1
Jcr4S00736.30	Aconitate hydratase (Aconitase)	B9T2U5	AT2G05710.1
Jcr4S00125.80	Succinyl-CoA synthetase	B9RL91	AT5G23250.1
Jcr4s06387.30	Succinyl-CoA synthetase	B9RL91	AT5G23250.1
Jcr4S03295.10	Malate dehydrogenase	B9SE47	AT1G53240.1
Jcr4S00279.20	Malate dehydrogenase	B9T5E4	AT5G43330.1
Jcr4s00055.90	Cytochrome C oxidase, putative	B9RV06	AT1G80230.1
Jcr4s01269.80	ATP synthase subunit beta	B9T1V8	AT5G08680.1
Jcr4s00914.20	ATP synthase subunit beta	B9T1V8	AT5G08680.1
Jcr4s00868.20	ATP synthase subunit gamma	B9SZS3	AT2G33040.1
Jcr4S01672.20	Ferredoxin	B9SCU0	AT2G27510.1
Jcr4S01794.30	Chlorophyll a-b binding protein	B9SID9	AT5G54270.1
Jcr4S01456.10	Phosphoglycerate dehydrogenase	B9RYA3	AT4G34200.1

We identified four cytosolic isoforms of glyceraldehyde-3-phosphate dehydrogenase (GAPDH) (Jcr4S07385.10, Jcr4S00953.40, Jcr4S00273.150, and Jcr4S00205.140) that catalyze the formation of 1,3-bisphosphoglycerate (BPGA) from Gly-3-P. In rapeseeds, the specific expression of GAPDH resulted in a 3–4-fold increase in G3P which ensured 40% improved oil deposition (Vigeolas et al., 2007). Such a role of GAPDH suggests that it might be the essential protein responsible for the significant amounts of oil deposition in an embryo. Additionally, we identified cytosolic isoform of phosphoglycerate mutase (Jcr4s28329.10) that was not identified in our previous studies from other seed tissues. It catalyzes the phosphate group interconversion between the C-3 carbon of 3-phosphoglycerate and C-2 carbon of 2-phosphoglycerate. Pyruvate, the outcome of glycolysis, serves as a precursor for acetyl-CoA. The production of acetyl-CoA primarily occurs through the pyruvate dehydrogenase complex (PDHC) inside the plastids (Joshee, 2006) and cannot penetrate the membrane. We have identified all the three subunits of plastidial PDHC (Jcr4S01924.60, Jcr4S00112.160, and Jcr4S04485.30) that support the biosynthesis of fatty acids within the plastids. All such proteins of the glycolytic pathway have already been reported from the endosperm and non-photosynthetic plastids of *J. curcas* seeds (Pinheiro et al., 2013; Shah et al., 2015). These consistent results support the notion that glycolytic pathway provides most of the carbon for FAS in growing embryos (Schwender et al., 2003).

Oxidative pentose phosphate pathway (OPPP) is another preferred route responsible for the generation of reducing power for fatty acids biosynthesis. We identified one oxidative phase protein PGL (Jcr4S16847.20) that catalyzes the hydrolysis of 6-phosphogluconolactone hydrolysis and one non-oxidative phase protein transketolase (Jcr4S00057.90) as well. It was observed that in sunflower, OPPP produces the bulk of reducing equivalents for the biosynthesis of fatty acids (Alonso et al., 2007). This study identified many functional glycolytic and OPPP proteins suggesting an active metabolism in the embryo.

Phosphoglucomutase (PGM) (Jcr4S00445.90) was identified here, catalyzing the reversible interconversion of Glc-1-P and Glc-6-P. The plastidial PGM is predominantly involved in the biosynthesis and degradation of starch molecules. Plastidial PGM mutant *Arabidopsis* seeds result in 40% less oil accretion in comparison with its wild-type seeds (Periappuram et al., 2000). Its cytosolic and plastidial isoforms have been reported in the endosperm (Shah et al., 2015), inner integument (Soares et al., 2014), and plastid of *J. curcas* seeds (Pinheiro et al., 2013). Identification of PGM establishes its significant impact on the accumulation of storage products in the seeds of *J. curcas*. We have identified cytosolic isoform of phosphoenolpyruvate carboxykinase (PEPCK) (Jcr4S08285.10), which catalyzes the synthesis of phosphoenolpyruvate from oxaloacetate. It has a vital role in the gluconeogenic production of sugar from stored oil during germination and early seedling growth in oilseed plants. Two PEPCK genes have been reported from *Arabidopsis* genome, both are believed to encode cytosolic proteins, with PEPCK1 being the prevalent gene expressed during early post-germinative growth (Rylott et al., 2001). Identification of PEPCK in our results suggests that this protein might have a crucial role in the provision of sugars during radical protrusion from embryo and seedling growth.

During the early phase of seed germination (oxygen deficient condition), the output of the TCA cycle is not sufficient and energy requirements are mostly met mainly by glycolysis and anaerobic respiration. In embryo, alcohol dehydrogenase is believed to be the part of glycol-metabolism where it catalyzes the synthesis of alcohol from pyranic acid reduction (Yang et al., 2007). Here, we have identified multiple isoforms of alcohol dehydrogenase including one unique isoform (Table 1) suggesting a major energy producer in hypoxia to facilitate the seed germination. As the energy from alcohol fermentation cannot fulfill the entire needs of germinating seeds. Therefore, at this point, TCA cycle produces the maximum amount of energy after the cellular environment is rich in oxygen (Miro et al., 2017). We identified three proteins of the TCA cycle, such as aconitate hydratase (Jcr4S09697.10), succinyl-CoA synthetase (Jcr4S00125.80), and malate dehydrogenase (Jcr4S03295.10). Although the TCA cycle is a major producer of energy during germination, the above three proteins identification hints that these proteins might be involved in reserves mobilization and provide energy for seedling growth. The identification of a significant number of the proteins related to carbohydrate metabolism indicates the importance of this pathway for energy production and providing precursors for lipid metabolism.

### Proteostasis and Defense Related Proteins

Many seeds can resist the environmental threats and develop into the new plant under favorable conditions. The emergence of adaptable strategies enables the seeds to defend themselves against stress. The failure can lead to the death of a new plant. Chaperones protect the proteins in their functionally active form and are concerned with assembly, folding and sustainability, and proteolysis (Trivedi et al., 2016). In this study, we identified 53 defense-related proteins (Table 3) corresponding to 9% of the total identified proteins. Eight isoforms of the chaperone (HSP70), an important class of chaperones associated with protein folding, and translocation to various cellular organelles, were identified. They inhibit protein accumulation and enable refolding of native proteins in normal and stress conditions as well. Additionally, we identified four isoforms of HSP 90 which are exclusively involved in the signaling pathways. Steroid hormone receptors and protein kinases are regarded as their substrates (Young et al., 2001).

**TABLE 3:** Proteostasis and defense related proteins identified from the embryo of mature *J. curcas* seeds.

Protein accession	Description	Best hit *Ricinus*	Best hit *Arabidopsis*
Jcr4s00582.50	Calreticulin (CRT)	B9RFI2	AT1G56340.2
Jcr4s03149.70	Calnexin (CNX)	B9RA41	AT5G61790.1
Jcr4s08787.20	Heat shock cognate 70 kDa protein	B9SR13	AT3G12580.1
Jcr4s00002.460	Heat shock 70 kDa protein	B9RGN3	AT1G16030.1
Jcr4s02598.30	Chaperone (mtHsc70)	B9RX55	AT5G09590.1
Jcr4s11847.20	Chaperone (Hsp70)	B9T228	AT5G02500.1
Jcr4s07413.10	Chaperone (Hsp70)	B9SP17	AT5G02500.1
Jcr4s03314.10	Chaperone (Hsp70)	B9T228	AT5G02500.1
Jcr4s01616.40	Chaperone (Hsp70)	B9RGN3	AT1G16030.1
Jcr4s01616.30	Chaperone (Hsp70)	B9RGN3	AT1G16030.1
Jcr4s00447.50	HSP70-chaperone (BiP)	B9SQC9	AT1G79930.1
Jcr4s03592.10	Chaperone (Hsp90)	B9T1Q8	AT5G56000.1
Jcr4s02234.80	Chaperone (Hsp90)	B9RIT0	AT5G52640.1
Jcr4s00535.40	Chaperone (Hsp90)	B9RCG8	AT5G52640.1
Jcr4s00006.110	Endoplasmin, putative (Hsp90)	B9R8A7	AT4G24190.1
Jcr4s04936.50	Chaperonin-60 kD, ch60, putative	B9S582	AT3G23990.1
Jcr4s00653.20	Chaperonin-60 kD	B9SBN5	AT3G13470.1
Jcr4s03954.10	Chaperonin-60 alpha	B9T7 × 8	AT2G28000.1
Jcr4s02414.50	Chaperonin CPN60-2, mitochondrial	B9RWQ2	AT3G23990.1
Jcr4s00940.30	Groes chaperonin, putative	B9SJ60	AT1G23100.1
Jcr4s00187.50	Chaperone protein ClpB1	B9RLP7	AT1G74310.1
Jcr4s00077.20	Nascent polypeptide associated complex alpha subunit, putative	B9SHV0	AT3G12390.1
Jcr4s01075.10	Heat shock 70 kDa protein, putative	B9S3M9	AT4G16660.1
Jcr4s02348.10	Nascent polypeptide associated complex alpha subunit, putative	B9S5M4	AT3G49470.1
Jcr4s07320.10	Jun activation domain binding protein, putative	B9SPP1	AT1G71230.1
Jcr4s00045.200	Ubiquitin, putative	B9RI09	AT5G20620.1
Jcr4s00168.60	Ubiquitin, putative	B9RI09	AT5G03240.3
Jcr4s00518.10	Ubiquitin, putative	B9RI09	AT4G05320.6
Jcr4s00540.60	Ubiquitin, putative	B9RI09	AT5G03240.3
Jcr4s01667.40	Ubiquitin, putative	B9RI09	AT1G31340.1
Jcr4s03519.90	Ubiquitin, putative	B9RI09	AT4G05050.3
Jcr4s04671.10	Ubiquitin, putative	B9RI09	AT1G31340.1
Jcr4s25965.10	Ubiquitin, putative	B9RI09	AT4G05050.3
Jcr4S02001.10	Ubiquitin	B9SWD7	AT3G52590.1
Jcr4S02833.30	Ubiquitin	B9RN74	AT2G47110.2
Jcr4S08473.50	Ubiquitin	B9SHC5	AT2G47110.2
Jcr4u30993.20	Small ubiquitin-related modifier (SUMO)	B9S510	AT4G26840.1
Jcr4s01038.60	Small ubiquitin-related modifier (SUMO)	B9S510	AT5G55160.1
Jcr4s00385.110	Regulatory component RPT5 of 26S proteasome	B9RFB5	AT3G05530.1
Jcr4s02802.40	20S Proteasome subunit alpha 2	B9RI05	AT1G16470.2
Jcr4s02462.10	E3 Ubiquitin-protein ligase	B9SMZ3	AT1G70320.1
Jcr4s00009.170	Transitional endoplasmic reticulum ATPase	B9RAY1	AT5G03340.1
Jcr4s01757.40	Transitional endoplasmic reticulum ATPase	B9S0I1	AT5G03340.1
Jcr4s02560.50	Transitional endoplasmic reticulum ATPase	B9S0I3	AT5G03340.1
Jcr4s00024.10	Class-C-I small heat-shock-responsive protein	B9S395	AT1G07400.1
Jcr4s00071.90	Class-M-I small heat-shock-responsive protein	B9SSG1	AT4G25200.1
Jcr4s01251.90	Class-C-I small heat-shock-responsive protein	B9S3B2	AT1G07400.1
Jcr4s04545.30	Class-C-I small heat-shock-responsive protein	B9S390	AT1G53540.1
Jcr4s10013.10	Class-C-I small heat-shock-responsive protein	B9S395	AT1G07400.1
Jcr4s11638.20	Class-C-I small heat-shock-responsive protein	B9SWN0	AT1G53540.1
Jcr4s18690.10	Class-C-I small heat-shock-responsive protein	B9S395	AT1G07400.1
Jcr4s02800.10	Class-C-I small heat-shock-responsive protein	B9S395	AT1G07400.1
Jcr4s03109.50	Class-M-II small heat-shock-responsive protein	B9RV59	AT1G52560.1

Further, four isoforms of HSP60 (Jcr4s04936.50, Jcr4s00653.20, Jcr4s03954.10, and Jcr4s02414.50) were identified which are ATP dependent mitochondrial chaperons. They are mainly involved in the import, refolding, and assembling of misfolded or unfolded proteins inside the mitochondrial matrix during stress conditions. In addition, these proteins facilitate the optimal growth and development of chloroplasts, embryos, and seedlings (Wang et al., 2004). Thus, the identification of various isoforms of heat shock proteins (HSPs) in the embryo of *J. curcas* seeds is indicative of the presence of the phenomenon of protein folding, translocation, and degradation in normal and stressed conditions.

The ubiquitin associated deterioration pathway plays a significant role in multiple aspects of plant growth. In the present study, the components of the ubiquitin proteasome system (UPS) were identified, such as isoforms of ubiquitin protein, 20S proteasome subunit alpha (Jcr4s02802.40), and regulatory component RPT5 of 26S proteasome (Jcr4s00385.110) (Table 3). E3 ubiquitin-protein ligase (Jcr4s02462.10) which acts as a modulator of plant responses to abiotic stresses, such as cold, heat, radiation, desiccation, salt, and nutrient deficiency were identified. The UPS promotes adaption to abiotic stress by managing the functioning of stress hormones like abscisic acid. It is accomplished through the activity of several ubiquitin ligases that control the signaling of various stress hormones (Komander and Rape, 2012). The identification of UPS in our data indicates the importance of this regulatory mechanism. It protects the embryo against environmental stress by degrading potentially harmful proteins, which monitor the concentration of key enzymes and regulatory proteins to maintain cellular homeostasis in the embryo of mature *J. curcas* seeds.

We identified eight sHSPs isoforms not identified previously from other tissues of *J. curcas* seeds (Table 3). These ubiquitous proteins are produced in response to a high temperature but are equally found during the particular phases of plant growth (Wehmeyer and Vierling, 2000). Here, the identification of multiple unique isoforms of sHSPs indicates that these proteins are embryo specific. They may have distinct regulatory controls and probably diverse functions during seed maturation. Additionally, they may be involved in protecting embryos during seeds desiccation tolerance, dormancy, and high temperature stress.

### Seed Storage Related Proteins

In *J. curcas* seeds, the nutrients are primarily deposited inside the endosperm and relatively less in the embryo itself (de Lopes et al., 2013). We identified 21 SSPs (Table 4) belonging to different classes. Five isoforms of legumins were identified which consist of six subunit pairs that interact non-covalently. Each subunit consists of an acidic (α-subunit 30–40 kDa) and a basic (β-subunit 20 kDa) unit joined covalently through a single disulfide bond. These chains are assembled within the protein bodies, yielding the mature forms and deposited in a particular temporal order with 7S (trimeric) and 11–12S globulins (hexameric) (Gruis et al., 2002). Six isoforms of 2S albumins including four embryo specific isoforms were identified here (Table 4). These are cysteine-containing water-soluble proteins found in a wide variety of dicotyledonous seeds having a protective role against fungus. The amino acids profile of these proteins from multiple plant species has shown a higher level of sulfur-containing amino acids (Moreno and Clemente, 2008) and identified as potential food allergens. Albumins were also reported from the endosperm (Shah et al., 2015) as well as the inner integument of *J. curcas* seeds (Soares et al., 2014).

**TABLE 4:** Seed storage related proteins identified from the embryo of mature *J. curcas*.

Protein accession	Description	Best hit *Ricinus*	Best hit *Arabidopsis*
Jcr4S00279.80	Legumin B, putative	B9T5E6	AT5G44120.3
Jcr4S01636.70	Legumin B, putative	B9SDX6	AT5G44120.4
Jcr4S15668.10	Legumin B, putative	B9SDX6	AT5G44120.1
Jcr4U29577.10	Legumin B, putative	B9SDX6	AT5G44120.3
Jcr4S01636.60	Legumin B, putative (Legumin-like protein)	Q9M4Q8	AT5G44120.3
Jcr4S01636.40	11S Globulin subunit beta, putative	B9SW16	AT1G03890.1
Jcr4S00619.40	2S Albumin, putative	B9SA33	AT5G54740.1
Jcr4S00619.90	2S Albumin, putative	B9SA28	AT5G54740.1
Jcr4S00279.60	Glutelin type-A 3, putative	B9T5E7	AT5G44120.3
Jcr4S03153.60	Nutrient reservoir, putative	B9SK34	AT4G36700.1
Jcr4S15278.20	Nutrient reservoir, putative	B9SK34	AT4G36700.1
Jcr4S17767.20	Vicilin GC72-A, putative	B9RTM9	AT3G22640.1
Jcr4S00353.50	Non-specific lipid-transfer protein, putative	B9SM06	AT3G18280.1
Jcr4S00033.50	Late embryogenesis abundant protein D-34, putative	B9RTR0	AT3G22490.1
Jcr4S02308.80	Late embryogenesis abundant protein D-34, putative	B9S3Z6	AT3G22490.1
Jcr4S02308.90	Late embryogenesis abundant protein D-34, putative	B9S3Z7	AT3G22490.1
Jcr4S05404.40	Late embryogenesis abundant protein D-7, putative	B9RV15	AT1G52690.2
Jcr4S01793.30	Late embryogenesis abundant protein Lea14-A, putative	B9T526	AT2G44060.2
Jcr4S00349.40	Late embryogenesis abundant protein, putative	B9RH90	AT2G21490.1
Jcr4S00025.50	Late embryogenesis abundant, putative	B9S010	AT2G18340.1
Jcr4S00300.40	Late embryogenesis abundant, putative	B9SRL2	AT2G36640.1
Jcr4S00706.60	Late embryogenesis abundant, putative	B9RBC1	AT4G21020.1
Jcr4S00619.50	Sweet protein mabinlin-1 chain A (Sweet protein mabinlin-1, chain B)	B9SA35	_
Jcr4S00619.60	Sweet protein mabinlin-1 chain A (Sweet protein mabinlin-1, chain B)	B9SA35	AT5G54740.1
Jcr4S00619.70	Sweet protein mabinlin-1 chain A (Sweet protein mabinlin-1, chain B)	B9SA35	AT4G27160.1
Jcr4S00619.80	Sweet protein mabinlin-1 chain A (Sweet protein mabinlin-1, chain B)	B9SA35	AT5G54740.1

We have identified two isoforms of vicilin-like SSPs (Jcr4S03153.60, Jcr4S15278.20) that belong to the cupin superfamily (nutrient reservoirs). They are highly diverse in the terms of polypeptide composition and involved in the plant defense response. These proteins have been reported from the embryo of *A. angustifolia* mature seeds, in which their accumulation was related to cotyledon differentiation and a defensive function against insect predation (de Sales et al., 2001).

Furthermore, we revealed the isoforms of antimicrobial proteins, such as vicilin-like (Jcr4s17767.20) and non-specific lipid-transfer proteins (nsLTPs) (Jcr4s20386.10 and Jcr4S00353.50). They exhibit antimicrobial activities because of their ability to permeabilize the cell membrane of phytopathogens (Scheurer and Schülke, 2018). This class of proteins was used as a biomarker to explore culture conditions during *Elaeis guineensis* somatic embryos maturation (Morcillo et al., 2001). The nsLTP was reported from the tomato endosperm and found to be involved in the transition of lipids from endosperm to embryo. They are also involved in the synthesis of a protective coating of cutin and suberin over the plant surface, and the defense against pathogens during seed germination (Scheurer and Schülke, 2018). Identifying these proteins in our analysis suggest their role in embryogenesis, seed maturation, lipid mobilization, signaling, and direct defense against pathogens during germination.

Besides these SSPs, we identified isoforms of the late embryogenesis abundant (LEA) (Table 4) proteins. These LEA proteins are synthesized during various phases of late embryogenesis in the seed embryo and under varying stress conditions, such as desiccation. The expression and deposition of LEA proteins in embryos indicate seed maturation and correlate water deficiency in various plant tissues and seed dehydration (Olvera-Carrillo et al., 2011). Since *J. curcas* seeds are orthodox type (desiccation resistant), LEA protein might be one of the major proteins contributing to the induction of this capacity to preserve seeds viability.

### Proteases

**FIGURE 3:**
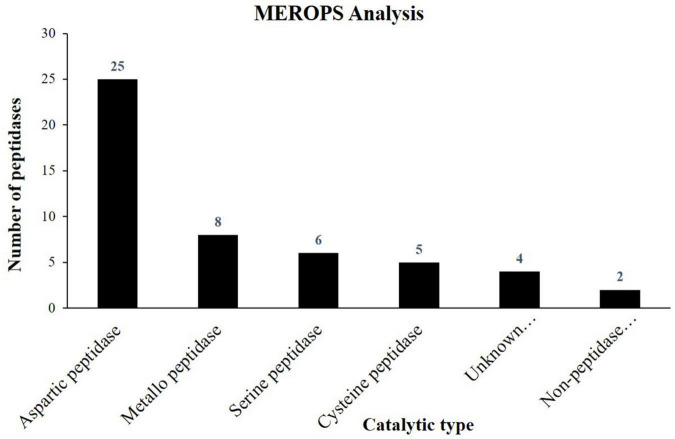
Column graph showing the catalytic types of peptidases identified from the embryo of *J. curcas* seeds in accordance to MEROPS database.

In the present study, 50 proteases and their inhibitors belonging to different mechanistic classes were identified (Table 5). Aspartic proteases (APs) are the most abundant class of peptidases, followed by Metallo (MPs), serine (SPs), and cysteine proteases (CPs) (Figure 3). APs were involved in the proteolysis and mobilization of reserve proteins (gliadin and globulin) in germinating wheat and rice seeds. In castor seeds, the involvement of APs in conjunction with vacuolar processing enzyme was proposed for the proteolytic processing and maturation of pro2S albumin pro-peptide (Hiraiwa et al., 1997). Six isoforms of 2S albumin protein were identified here (Table 4) which indicate that in the embryo of *J. curcas* these APs might be involved in their maturation. It was also hypothesized that during PCD, APs could be the part of nucellar cells deterioration and be involved in the synthesis of new proteins from nucellar cell death proteins for the growth of embryo and endosperm in barley (Chen and Foolad, 1997). Previously, we identified six homologs of *Arabidopsis* phytepsins from the endosperm of *J. curcas* seeds (Shah et al., 2015), however, in the present study only one isoform of phytepsin (Jcr4S00547.20) was identified, suggesting that similar isomers of the enzyme may be active in different tissues of this plant. The identification of APs in our data suggests their multiple roles, such as maturation of reserves, mobilization, and proteolytic hydrolysis of stored reserves during seed germination and in PCD.

**TABLE 5:** A list of peptidases and their inhibitors identified from the embryo of mature *J. curcas* seeds.

Protein accession	MEROPS ID	Description	Catalytic type
Jcr4S00063.130	MER412334	Aspartic proteinase, putative	Aspartic
Jcr4S00107.20	MER178448	Uncharacterized protein	Aspartic
Jcr4S00547.20	MER496613	Aspartic proteinase (phytepsin) putative	Aspartic
Jcr4S00739.60	MER170824	Aspartic proteinase nepenthesin-1, putative	Aspartic
Jcr4S01204.30	MER200104	Uncharacterized protein	Aspartic
Jcr4S01708.30	MER200104	Uncharacterized protein	Aspartic
Jcr4S02365.20	MER177024	Poly [ADP-ribose] polymerase (PARP)	Aspartic
Jcr4S02367.30	MER168322	CCHC-type domain-containing protein	Aspartic
Jcr4S02531.20	MER200104	Uncharacterized protein	Aspartic
Jcr4S03044.70	MER200104	Uncharacterized protein	Aspartic
Jcr4S06099.10	MER200104	Uncharacterized protein	Aspartic
Jcr4S06992.10	MER165268	Chaperone binding protein, putative	Aspartic
Jcr4S07071.50	MER161139	CCHC-type domain-containing protein	Aspartic
Jcr4S07203.10	MER165958	Uncharacterized protein	Aspartic
Jcr4S08659.10	MER200073	_	Aspartic
Jcr4S09276.10	MER199445	Uncharacterized protein	Aspartic
Jcr4S09731.30	MER201208	Uncharacterized protein	Aspartic
Jcr4S10915.10	MER200446	Retrotrans gag domain-containing protein	Aspartic
Jcr4S11449.10	MER176399	Uncharacterized protein	Aspartic
Jcr4S16426.20	MER199779	Uncharacterized protein	Aspartic
Jcr4S16499.10	MER164887	Uncharacterized protein	Aspartic
Jcr4S19565.30	MER200104	Uncharacterized protein	Aspartic
Jcr4S20272.10	MER199779	Uncharacterized protein	Aspartic
Jcr4S27524.10	MER199659	Glyceraldehyde-3-phosphate dehydrogenase	Aspartic
Jcr4S28308.20	MER199779	Uncharacterized protein	Aspartic
Jcr4S00009.170	MER269263	Transitional endoplasmic reticulum ATPase, putative	Metallo
Jcr4S00039.10	MER413614	Transitional endoplasmic reticulum ATPase, putative	Metallo
Jcr4S00343.100	MER172798	Leucine aminopeptidase, putative	Metallo
Jcr4S00385.110	MER278187	26S Protease regulatory subunit 6a, putative	Metallo
Jcr4S01168.90	MER172955	Oligopeptidase A (TOP1 peptidase), putative	Metallo
Jcr4S01757.40	MER413614	Transitional endoplasmic reticulum ATPase, putative	Metallo
Jcr4S02560.50	MER278187	Transitional endoplasmic reticulum ATPase, putative	Metallo
Jcr4S07320.10	MER143437	Jun activation domain binding protein, putative	Metallo
Jcr4S00788.120	MER157367	WD-repeat protein, putative	Serine
Jcr4S00899.60	MER178907	Transporter, putative	Serine
Jcr4S00913.60	MER146853	Nucleotide binding protein, putative	Serine
Jcr4S01323.70	MER135263	WD-repeat protein, putative	Serine
Jcr4S01500.30	MER170587	Tripeptidyl-peptidase II	Serine
Jcr4S02309.30	MER171392	Xylem serine proteinase 1, putative	Serine
Jcr4S00724.60	MER038019	Stem-specific protein TSJT1, putative	Cysteine
Jcr4S01609.40	MER158866	Cysteine protease (RD21), putative	Cysteine
Jcr4S02110.50	MER178955	Caspase (metacaspase), putative	Cysteine
Jcr4S04275.10	MER126050	Uncharacterized protein	Cysteine
Jcr4S16229.10	MER292732	Cysteine protease, putative	Cysteine
Jcr4S00079.140	MER180116	Protein Z (AtSerpin1), putative	Unknown
Jcr4S02989.80	MER020315	Cysteine proteinase inhibitor (phytocystatin)	Unknown
Jcr4S17142.10	MER169281	Cysteine proteinase inhibitor B (cystatin), putative	Unknown
Jcr4S28633.30	MER200074	Uncharacterized protein	Unknown
Jcr4S02802.40	MER170530	Proteasome subunit alpha type	Non-peptidase homolog
Jcr4S02821.10	MER176444	Proliferation-associated 2g4, putative	Non-peptidase homolog

We identified eight MPs including *Arabidopsis* homolog of thimet oligopeptidase known as TOP1 peptidase (Jcr4S01168.90), constituting a class of salicylic acid (SA) binding proteins (Moreau et al., 2013). As SA plays important role in plant immune response, its identification in our data suggests its interaction with SA. It may play important role in defense mechanisms to protect the embryo from various types of biotic and abiotic stresses. FtsH endopeptidases were identified that are ATP-dependent zinc MPs and are embryo-specific associated with a broad range of cellular functions. The identification of MPs indicates that these proteins might be responsible for the rapid turnover and stability of large protein complexes to maintain cellular homeostasis inside the embryo of *J. curcas* seeds.

In addition, five CPs were identified here (Table 5) including 2 papain like proteases (Jcr4S01609.40 and Jcr4S16229.10). As seeds are the major sites for structural, metabolic, defensive, and reserve proteins, PLCPs are primarily involved in the mobilization and degradation of these proteins during seed germination. In the growing seedlings of wheat and maize, CPs account for 90% of the overall protease activity of prolamins (Grudkowska and Zagdańska, 2004). During germination, seedling requires nutrients and energy, provided by the seed reserves. In this context, the identification of these proteases indicates their involvement in the mobilization and degradation of reserves during seed germination.

Plants lack caspases homologs but contain a phylogenetically distinct class of CPs known as metacaspases. These are also identified in our data (Table 5). Although the exact function of metacaspases in the PCD mechanism is still unclear, their differential expression was found to affect the PCD in plant embryos (Suarez et al., 2004). Thus, the identification of metacaspase indicates its significance in PCD during embryonic pattern formation and also presents a link between PCD and plant embryogenesis. Furthermore, a previous study reported that KDEL-tailed cysteine peptidases along with vacuolar processing enzymes (VPEs) were responsible for the occurrence of PCD in the endosperm (Shah et al., 2015) and inner integument (Soares et al., 2014) of *J. curcas* seeds. These results collectively reveal the involvement of a variety of peptidases in PCD in the different tissues of *J. curcas* seeds.

Additionally, we identified proteinase inhibitors (PIs), such as two cysteine protease inhibitors, namely, cystatins/phytocystatins (Jcr4S02989.80 and Jcr4S17142.10) and one serine protease inhibitor, namely, serpin (Jcr4S00079.140) (Table 5). Cystatin is actively produced in growing seeds and vegetative storage tissues to outnumber CPs and support reserve proteins. A high cystatin/CPs balance retained in quiescent tissues enables the pool of stored proteins to be preserved over dormancy and made available upon seed germination. After seed imbibition, the upregulation of CPs encoding genes while the downregulation of cystatin encoding genes causes a sharp decrease in cysteine/cystatin protease balance which favors the mobilization and hydrolysis of stored proteins in amaranth and *Arabidopsis* (Hwang et al., 2009). The identification of cystatin in this study suggests its role in regulating CPs activity to prevent an unscheduled hydrolysis of SSPs as well as protecting the embryo from the exogenous CPs of phytopathogens. Serpins inhibit cysteine proteases specially RD21 cysteine protease (Jcr4S01609.40) which is also identified here. Such proteins are concerned with desiccation response and pathogenic defense (Lampl et al., 2010). Serpin and RD21 cysteine protease were identified from the endosperm of *J. curcas* seeds (Shah et al., 2015). The identification of serpin in our data reveals its role to retain RD21 inactive until required during germination.

### Transport Related Proteins

**FIGURE 4:**
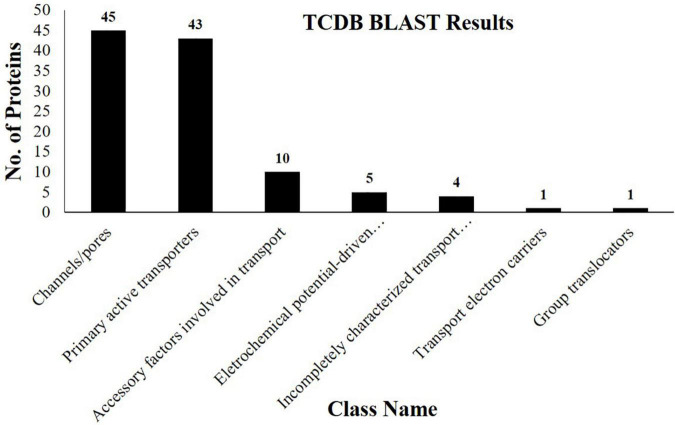
Column graph showing the various classes of transporter proteins identified from the embryo of *J. curcas* seeds in accordance with the Transporter Classification Database (TCDB).

Besides other important proteins, we identified 109 different transport-related proteins corresponding to almost 19% of the total identifications (Supplementary Table 2). Based on TCDB database information, these proteins belong to seven different classes. Channel/pores are the most representative class of transporters identified, followed by primary active transporters, accessory factors involved in transport, electrochemical potential-driven transporters, incompletely characterized transport systems, transport electron carriers, and group translocators (Figure 4). We identified 45 members of the channels/pores family of transporters (Supplementary Table 2). Among them, 10 transporters belong to the family of HSPs (HSP70) which are abundantly present in multiple living species. They are responsible for the transport of proteins in different cellular organelles and in translocating misfolded proteins toward the UPS for deterioration. They are also involved in the transport of many transmembrane proteins, aids in their folding, and protect them from stressful conditions (Trivedi et al., 2016). The identification of HSP70 in our study indicates their involvement in the translocation of misfolded and other functional proteins toward their target site to maintain cellular homeostasis in normal and stressful conditions. We identified Aquaporin PIP2 (Jcr4S02148.40), a channel protein that belongs to the membrane intrinsic protein family. They promote the passive movement of water and other solute molecules through the membrane, facilitated by osmotic or solute gradients. Genes coding for PIP along with other Aquaporin, i.e., PIP2, TIP1 were found to express in cotyledons and seed coats of growing pea seeds (Schuurmans et al., 2003). Though information regarding the functioning of PIPs during seed maturation is not yet available, however, 1l Aquaporins have been reported from the dry seeds of *Arabidopsis* (Vander Willigen et al., 2006). They are supposed to be pre-formed during the growth and maturation of seeds. Since mature seeds are dried and require water for germination, the identification of Aquaporin in our study suggests that they might be responsible for the transport of water and probably other solute molecules in the embryo to facilitate efficient seed germination.

Five members of electrochemical potential-driven transporters were identified here (Supplementary Table 2). They include amino acid transporters isoforms (Jcr4S09615.20 and Jcr4S07701.10), previously not identified from this species. These transporters are important for the transport of amino acids to cells and organelles, such as mitochondria, peroxisomes, chloroplast, and vacuole to ensure an equal supply of organic nitrogen (Dinkeloo et al., 2018). In addition, studies indicated the important functions of various transporters, i.e., AAP1 and AAP8 in *Arabidopsis* during the embryonic distribution of amino acids to promote the seed growth and reserves deposition. Among them, particularly AAP1 was found to be involved in the transfer of amino acids toward the embryo. AAP1 deficient embryos revealed the lower import of amino acids leading to the lower accumulation of reserve proteins (Sanders et al., 2009). Similarly, it was hypothesized that the upregulation of AAP1 encoding gene ensued increased deposition of reserves in pea seeds broad beans (Miranda et al., 2001). The presence of amino acid transporter indicates that these may be embryo-specific and present a positive correlation with the accumulation of SSPs identified here. The appearance of these proteins indicates their role in regulating the synthesis of SSPs and reveals their importance for the completion of the nitrogen cycle in seeds.

## Conclusion

In this study, we presented a proteome analysis of the embryo isolated from the mature *J. curcas* seeds. This analysis resulted in the identification of 564 proteins of which 206 proteins were not identified from other tissue of this plant so far. We were able to identify the proteins responsible for the provision of carbon source and energy, including but not limited to carbohydrate metabolism, lipids metabolism, transport-related proteins, and proteases of different mechanistic classes. In the case of *J. curcas* seeds, nutrients are primarily deposited inside the endosperm and relatively less in the embryo. However, the identification of several classes of the SSPs, including the members of legumins and vicilins as well as the transporters, such as amino acids transporters indicates the potential of this tissue for supporting the germination process in the terms of the provision of nutrients. Our analysis furnishes insight into the important pathways and certain unique features of the embryo from the mature seeds of *J. curcas*. The findings presented here are a step forward in our efforts to create a proteome catalog of *J. curcas* seeds, which will serve as a significant resource for studies on the developmental biology of oilseeds.

## Data Availability Statement

The original contributions presented in the study are publicly available. This data can be found here: Mass spectrometry raw data files are available on the Chorus repository web site (chorusproject.org; project ID: 1747, project name: *Jatropha curcas* Embryo Proteome).

## Author Contributions

MS, MA, and FN designed the experiment and wrote the manuscript. AR, NU, Sheheryar, and JN performed the experiments and analyzed the data. FC and GD proofread the manuscript. All authors read and approved of its content, read and agreed to the published version of the manuscript.

## Conflict of Interest

The authors declare that the research was conducted in the absence of any commercial or financial relationships that could be construed as a potential conflict of interest.

## Publisher’s Note

All claims expressed in this article are solely those of the authors and do not necessarily represent those of their affiliated organizations, or those of the publisher, the editors and the reviewers. Any product that may be evaluated in this article, or claim that may be made by its manufacturer, is not guaranteed or endorsed by the publisher.

## Footnotes

1
http://www.arabidopsis.org/

2
http://www.uniprot.org/

3
https://mapman.gabipd.org/

4
https://www.ebi.ac.uk/merops/

5
http://www.tcdb.org/

## Supplementary Material

Supplementary Table 1 A list of proteins and peptides identified from the proteome analysis of the embryo isolated from mature *Jatropha curcas* seeds.
