# Supplementary material for: Proteomic Analysis of Embryo Isolated From Mature Jatropha curcas L. Seeds
Source: Front Plant Sci. 2022 Mar 18;13:843764. doi: 10.3389/fpls.2022.843764 (PMC8971811; doi:10.3389/fpls.2022.843764)
Supplement: Supplementary file 2 [file Table_2.DOCX]

**Supplementary Table II**. List of transporter proteins identified from the embryo of mature *Jatropha curcas* seeds.

| **Protein Accession** | **Description** | **Class Name** | **Class** |
| --- | --- | --- | --- |
| Jcr4S00739.60 | Aspartic proteinase nepenthesin-1, putative | Channels/pores | 10600.1 |
| Jcr4S00240.130 | Glutamate receptor | -do- | 1.A.10.1.10 |
| Jcr4S00003.380 | Translocation protein SEC62 | -do- | 1.A.15.1.4 |
| Jcr4S00482.30 | NB-ARC domain-containing protein | -do- | 1.A.25.3.4 |
| Jcr4S03588.20 | Disease resistance protein RGA2 | -do- | 1.A.25.3.4 |
| Jcr4S00002.460 | Heat shock protein, putative | -do- | 1.A.33.1.1 |
| Jcr4S00447.50 | Heat shock 70 kDa protein, putative | -do- | 1.A.33.1.1 |
| Jcr4S01616.30 | Heat shock protein, putative | -do- | 1.A.33.1.1 |
| Jcr4S01616.40 | Heat shock protein, putative | -do- | 1.A.33.1.1 |
| Jcr4S03314.10 | Heat shock protein, putative | -do- | 1.A.33.1.1 |
| Jcr4S07413.10 | Heat shock protein, putative | -do- | 1.A.33.1.1 |
| Jcr4S08787.20 | Heat shock protein, putative | -do- | 1.A.33.1.1 |
| Jcr4S11847.20 | Heat shock protein, putative | -do- | 1.A.33.1.1 |
| Jcr4S02598.30 | Heat shock protein, putative | -do- | 1.A.33.1.2 |
| Jcr4S01075.10 | Heat shock 70 kDa protein, putative | -do- | 1.A.33.1.3 |
| Jcr4S07929.20 | Formin-like protein | -do- | 1.A.33.1.6 |
| Jcr4S02004.20 | Phosphoprotein phosphatase, putative | -do- | 1.A.51.2.1 |
| Jcr4S02148.40 | Aquaporin (Aquaporin PIP2.7, putative) | -do- | 1.A.8.11.6 |
| Jcr4S02203.10 | ATP binding protein, putative | -do- | 1.A.87.2.1 |
| Jcr4S20081.10 | ATP binding protein, putative | -do- | 1.A.87.2.3 |
| Jcr4S01532.40 | Sorting and assembly machinery (Sam50) protein, putative | -do- | 1.B.33.2.1 |
| Jcr4S00603.20 | Acc synthase, putative | -do- | 1.C.121.1.1 |
| Jcr4S03723.20 | Nucleolar protein nop56, putative | -do- | 1.C.121.1.1 |
| Jcr4S17767.20 | Vicilin GC72-A, putative | -do- | 1.C.121.1.1 |
| Jcr4S00279.60 | Glutelin type-A 3, putative | -do- | 1.C.121.1.2 |
| Jcr4S00279.80 | Legumin B, putative | -do- | 1.C.121.1.2 |
| Jcr4S01636.40 | 11S globulin subunit beta, putative | -do- | 1.C.121.1.2 |
| Jcr4S01636.60 | Legumin B, putative (Legumin-like protein) | -do- | 1.C.121.1.2 |
| Jcr4S01636.70 | Legumin B, putative | -do- | 1.C.121.1.2 |
| Jcr4S15668.10 | Legumin B, putative | -do- | 1.C.121.1.2 |
| Jcr4U29577.10 | Legumin B, putative | -do- | 1.C.121.1.2 |
| Jcr4S04669.10 | MACPF domain-containing protein | -do- | 1.C.39.11.2 |
| Jcr4S00899.60 | Transporter, putative | -do- | 1.I.1.1.1 |
| Jcr4S00005.310 | Myosin XI, putative | -do- | 1.I.1.1.3 |
| Jcr4S00161.60 | Eukaryotic translation initiation factor 5A (eIF-5A) | -do- | 1.I.1.1.3 |
| Jcr4S00232.50 | Uncharacterized protein | -do- | 1.I.1.1.3 |
| Jcr4S02143.40 | Eukaryotic translation initiation factor 5A (eIF-5A) | -do- | 1.I.1.1.3 |
| Jcr4S02223.40 | Dead box ATP-dependent RNA helicase, putative | -do- | 1.I.1.1.3 |
| Jcr4S02821.20 | Ran binding protein, putative | -do- | 1.I.1.1.3 |
| Jcr4S03493.70 | Eukaryotic translation initiation factor 5A (eIF-5A) | -do- | 1.I.1.1.3 |
| Jcr4S00313.50 | (1->3)-beta-glucan endohydrolase (Beta-1,3-endoglucanase) | -do- | 1.I.2.1.1 |
| Jcr4S00587.110 | Hydrolase, hydrolyzing O-glycosyl compounds, putative | -do- | 1.I.2.1.1 |
| Jcr4S02325.10 | (1->3)-beta-glucan endohydrolase (Beta-1,3-endoglucanase) | -do- | 1.I.2.1.1 |
| Jcr4S09288.30 | Hydrolase, hydrolyzing O-glycosyl compounds, putative | -do- | 1.I.2.1.1 |
| Jcr4S27076.10 | (1->3)-beta-glucan endohydrolase (Beta-1,3-endoglucanase) | -do- | 1.I.2.1.1 |
| Jcr4S02827.30 | Spermidine synthase 1, putative | -do- | 2.A.1.86.5 |
| Jcr4S09615.20 | Amino acid transporter, putative | -do- | 2.A.18.8.4 |
| Jcr4S01609.40 | Cysteine protease, putative | -do- | 2.A.28.3.6 |
| Jcr4S16229.10 | Cysteine protease, putative | -do- | 2.A.28.3.6 |
| Jcr4S07701.10 | Amino acid transporter, putative | -do- | 2.A.3.12.3 |
| Jcr4S00009.170 | Transitional endoplasmic reticulum ATPase, putative | Primary active transporters | 3.A.16.1.1 |
| Jcr4S00039.10 | Transitional endoplasmic reticulum ATPase, putative | -do- | 3.A.16.1.1 |
| Jcr4S01757.40 | Transitional endoplasmic reticulum ATPase, putative | -do- | 3.A.16.1.1 |
| Jcr4S02560.50 | Transitional endoplasmic reticulum ATPase, putative | -do- | 3.A.16.1.1 |
| Jcr4S00045.200 | Ubiquitin, putative | -do- | 3.A.16.1.3 |
| Jcr4S00050.80 | Ubiquitin, putative | -do- | 3.A.16.1.3 |
| Jcr4S00168.60 | Ubiquitin, putative | -do- | 3.A.16.1.3 |
| Jcr4S00385.110 | 26S protease regulatory subunit 6a, putative | -do- | 3.A.16.1.3 |
| Jcr4S00518.10 | Ubiquitin, putative | -do- | 3.A.16.1.3 |
| Jcr4S00540.60 | Ubiquitin, putative | -do- | 3.A.16.1.3 |
| Jcr4S02833.30 | Ubiquitin | -do- | 3.A.16.1.3 |
| Jcr4S02914.10 | Ubiquitin | -do- | 3.A.16.1.3 |
| Jcr4S03519.90 | Ubiquitin, putative | -do- | 3.A.16.1.3 |
| Jcr4S08667.20 | Ubiquitin | -do- | 3.A.16.1.3 |
| Jcr4S25965.10 | Ubiquitin, putative | -do- | 3.A.16.1.3 |
| Jcr4S00005.10 | Dead box ATP-dependent RNA helicase, putative | -do- | 3.A.18.1.1 |
| Jcr4S00285.110 | Dead box ATP-dependent RNA helicase, putative | -do- | 3.A.18.1.1 |
| Jcr4S02656.20 | SAP domain-containing protein | -do- | 3.A.18.1.1 |
| Jcr4S00868.20 | ATP synthase subunit gamma | -do- | 3.A.2.1.11 |
| Jcr4S00914.20 | ATP synthase subunit beta | -do- | 3.A.2.1.11 |
| Jcr4S01269.80 | ATP synthase subunit beta | -do- | 3.A.2.1.11 |
| Jcr4S00089.140 | Peroxin-14 | -do- | 3.A.20.1.2 |
| Jcr4S00409.80 | Heat shock protein 70 (HSP70)-interacting protein, putative | -do- | 3.A.21.1.1 |
| Jcr4S00836.20 | THO complex subunit 2 | -do- | 3.A.22.1.1 |
| Jcr4S00100.140 | Glyceraldehyde-3-phosphate dehydrogenase | -do- | 3.A.23.6.1 |
| Jcr4S00205.140 | Glyceraldehyde-3-phosphate dehydrogenase | -do- | 3.A.23.6.1 |
| Jcr4S00273.150 | Glyceraldehyde-3-phosphate dehydrogenase | -do- | 3.A.23.6.1 |
| Jcr4S27524.10 | Glyceraldehyde-3-phosphate dehydrogenase | -do- | 3.A.23.6.1 |
| Jcr4U29393.10 | Glyceraldehyde-3-phosphate dehydrogenase | -do- | 3.A.23.6.1 |
| Jcr4S01667.40 | Ubiquitin, putative | -do- | 3.A.25.1.1 |
| Jcr4S02001.10 | Ubiquitin | -do- | 3.A.25.1.1 |
| Jcr4S04671.10 | Ubiquitin, putative | -do- | 3.A.25.1.1 |
| Jcr4S08473.50 | Ubiquitin | -do- | 3.A.25.1.1 |
| Jcr4S01614.80 | Adenosinetriphosphatase | -do- | 3.A.28.1.3 |
| Jcr4S01772.110 | Protein transport protein Sec61 subunit beta | -do- | 3.A.5.4.2 |
| Jcr4S00593.120 | Sec61 gamma subunit, putative | -do- | 3.A.5.9.1 |
| Jcr4S00913.60 | Nucleotide binding protein, putative | -do- | 3.A.5.9.1 |
| Jcr4S03684.40 | Signal recognition particle subunit SRP72 | -do- | 3.A.5.9.1 |
| Jcr4S00187.50 | Chaperone clpb, putative | -do- | 3.A.9.1.2 |
| Jcr4S00154.140 | Peptidyl-prolyl cis-trans isomerase (PPIase) | -do- | 3.D.1.8.1 |
| Jcr4S01173.10 | Peptidyl-prolyl cis-trans isomerase (PPIase) | -do- | 3.D.1.8.1 |
| Jcr4S00055.90 | Cytochrome C oxidase, putative | -do- | 3.D.4.11.1 |
| Jcr4S00609.30 | Alcohol dehydrogenase, putative | -do- | 3.D.4.8.1 |
| Jcr4S01497.10 | AMP dependent CoA ligase, putative | Group translocators | 4.C.1.1.7 |
| Jcr4S01794.30 | Chlorophyll a-b binding protein, chloroplastic | Transport electron carriers | 5.B.4.1.1 |
| Jcr4S03363.60 | Reticulon-like protein | Accessory factors involved in transport | 8.A.102.1.1 |
| Jcr4S08573.20 | Peptidylprolyl isomerase | -do- | 8.A.11.1.1 |
| Jcr4S01421.20 | Peptidylprolyl isomerase | -do- | 8.A.11.1.2 |
| Jcr4S01799.20 | Peptidylprolyl isomerase | -do- | 8.A.11.1.2 |
| Jcr4S08573.10 | Peptidylprolyl isomerase | -do- | 8.A.11.1.2 |
| Jcr4S00063.130 | Aspartic proteinase, putative | -do- | 8.A.32.1.4 |
| Jcr4S00547.20 | Aspartic proteinase, putative | -do- | 8.A.32.1.4 |
| Jcr4S04499.20 | Calmodulin | -do- | 8.A.82.1.1 |
| Jcr4S07226.30 | Calmodulin | -do- | 8.A.82.1.1 |
| Jcr4S06094.20 | Protein disulfide-isomerase | -do- | 8.A.88.1.3 |
| Jcr4S01215.100 | GTP-binding nuclear protein | Incompletely characterized transport systems | 9.A.50.1.1 |
| Jcr4S02912.10 | GTP-binding nuclear protein | -do- | 9.A.50.1.1 |
| Jcr4S06442.10 | Peroxidase | -do- | 9.A.61.3.1 |
| Jcr4S09680.10 | Zinc finger protein, putative | -do- | 9.B.87.1.6 |
